# Participation in Project ECHO to advance rural primary care providers’ ability to address patient mental health needs

**DOI:** 10.1080/10872981.2022.2164470

**Published:** 2023-01-02

**Authors:** Sonya Panjwani, Ariel Porto, Rosemary Motz, Maximillian Morris, Lynsey Grzejszczak, Anthony Dimartino, Karen Ashley

**Affiliations:** Weitzman Institute, Community Health Center, Inc, Middletown, CT, USA

**Keywords:** Continuing education, rural primary care, Project ECHO, adult psychiatry, mental health

## Abstract

The COVID-19 pandemic shed light on the burden of behavioral health conditions prevalent in the United States (U.S.). Consequently, there is a behavioral healthcare provider shortage, particularly in rural areas, to support this need. Recently, primary care providers (PCPs) have shifted to incorporate behavioral health to their practice. However, many PCPs lack knowledge and skills to successfully manage their patients’ behavioral health conditions. In response to the need for effective behavioral healthcare across the U.S. Weitzman ECHO launched the Advanced Primary Care (APC ECHO) Adult Psychiatry Module to provide continuing education (CE) for rural PCPs. This study presents the results from the APC ECHO pilot to demonstrate how CE can support PCPs in addressing their patients’ mental health needs. Evaluators used a one-group repeated measures study design to assess the APC ECHO Module and understand learner outcomes and individual practice changes. Participant characteristics and individual practice changes were summarized using descriptive statistics, with support from open-ended responses to illustrate findings. Repeated measures analyses of covariance were applied to compare the differences in pre- and post-module learner outcomes. A total of 18 providers participated in the study, with the majority encompassing medical providers (72.2%). There was a significant increase in knowledge (pre-module: 21.11 + 6.99; post-module: 25.08 + 5.66; *p* < .01), self-efficacy (pre-module: 6.89 + 3.05; post-module: 9.78 + 3.25; *p* < .01), and skills (pre-module: 7.67 + 4.03; post-module: 10.06 + 3.23; *p* < .05) gained over the duration of the ECHO module. Additionally, participants indicated they are applying best practices learned through the module to their patients experiencing psychiatric conditions (3.96 + 0.09). This study suggests that tailored CE for PCPs can promote an increase in knowledge, self-efficacy, and skills to apply best practices when treating patients with behavioral health conditions. This, in turn, allows patients to receive more comprehensive care and mitigates access barriers, especially for rural populations.

## Introduction

Behavioral health conditions affected more than 1 billion people globally in 2016 [1], with variations based on gender, minority status, socioeconomic status, among other distinguishing factors. For example, research indicates that women experience higher rates of mood disorders, while men experience higher rates of substance use disorders [2]. For the purposes of this article, we define behavioral health as the emotions and behaviors that affect overall well-being, and the terms behavioral and mental health will be used interchangeably [3]. In 2020, the onset of the COVID-19 pandemic exacerbated the burden of mental health disorders in the United States (U.S.), leading to an estimated 52.9 million adults experiencing at least one mental health disorder; among those identified, only about half (24.3 million) received any sort of mental health service in the prior year [4]. This startling lack of treatment for mental health conditions is often much higher in rural counties in the U.S., where there is a severe lack of psychiatrists and psychologists compared to urban counties [5]. When behavioral health providers are present in these rural areas, they are often situated separately from primary care providers, with only 26% of primary care locations in rural areas integrating a behavioral health provider. This translates to greater barriers faced by rural residents due to the burden of having to travel long distances when seeking mental healthcare [6]. Furthermore, in addition to the lack of mental health providers, mental health facilities in rural areas often do not accept Medicaid or state insurance, which typically provides coverage for a large portion of rural populations [7].

In keeping with the access barriers for mental healthcare in rural areas, the option of receiving behavioral health services from a primary care provider often becomes the only solution to meet the mental healthcare needs of rural populations. Research suggests the benefits of having a primary care provider deliver behavioral health treatments include an already existing and strong patient/provider relationship, convenience for patients, and patient willingness to accept mental healthcare through a trusted source, which may reduce barriers related to stigma around mental health services [8]. However, many primary care providers experience a gap in their knowledge in how to successfully manage their patients with behavioral health conditions. In a recent study of 241 family physicians, 58.1% reported that they had either no or marginal knowledge surrounding psychotropic medications [9]. Patients have also reported that the lack of providers’ ability to counsel them created a barrier to receiving behavioral healthcare; in most instances, patients only received medication for their condition [8].

Tailored Continuing Education (CE) training that is easily accessible to support primary care providers in their treatment of patients for behavioral health conditions becomes crucial to close gaps in knowledge, confidence, and skills. Of the 241 family physicians in the previously cited report, 75.1% reported that they were quite willing and interested in learning more about psychotropic medications to help their patients [9]. Thus, developing targeted support through CE training for primary care providers to learn from behavioral health specialists is critical in improving provider confidence, treatment plans, and patient experiences [8].

### Weitzman ECHO program

As a response to the extensive need for effective mental health treatment across the U.S., compounded by the disparities in access experienced in rural areas, the Community Health Center, Inc. in Connecticut, through its Weitzman Institute (WI), developed a multi-component educational intervention utilizing the proven Project ECHO® telementoring model [10]. Project ECHO® is a virtual, case-based CE model designed to expand the capacity of a target audience of professionals in a selected focus area through ongoing access to an engaged learning community of experts and peers. As of August 2022, Project ECHO® has been deployed internationally in 59 countries by 792 organizational ‘hubs’ [11].

As the first Federally Qualified Health Center (FQHC) to replicate Project ECHO® in 2012, WI has a long history of implementing Project ECHO® programs to support providers caring for underserved patients. As of September 2022, over 8,000 providers and care team members from all 50 states, Washington D.C., and Puerto Rico have participated across 21 unique WI Project ECHO (Weitzman ECHO) programs. Each Weitzman ECHO session provides a brief (20–25 minutes) formal didactic presentation by a team of multidisciplinary experts in a specific topic relative to the program’s focus area, followed by one or more live case presentations from participants who encompass providers or care team members. After the case presentation, faculty experts facilitate a discussion of recommendations to educate participants and improve their ability to manage complex patient cases more effectively [12].

The present project was developed with a grant through the Health Resources and Services Administration (HRSA) Office for the Advancement of Telehealth’s Telehealth Technology-Enabled Learning Program (TTELP), which focuses on increasing primary care providers’ access to specialist expertise in rural, frontier, and underserved communities in the U.S. WI’s TTELP project comprises two distinct ECHO programs to equip providers with the knowledge, skills, and confidence to manage complex cases across the lifespan to address these communities; this study focuses specifically on the adult patient population through the Weitzman ECHO Advanced Primary Care (APC ECHO). In year one of the project, the APC ECHO held two modules of 8 sessions each, with the first focusing on adult psychiatry and the second on substance use disorders. The program was led by relevant subject matter experts of ‘core faculty’ who attended and guided each session, including four experienced primary care physicians who manage complex patient panels at FQHCs, one behavioral health clinician, and one psychiatrist.

The APC ECHO was developed to provide targeted support in the selected, in-demand topic areas for providers and care team members in rural settings across the country but were also open to those outside of rural areas serving underserved populations. Participants were recruited primarily through the WI’s national email network filtered by rural zip codes and states with large rural areas, with additional outreach through partnering FQHCs and organizations. Participants were welcome from any area of the country as long as they provided direct care to patients or support to providers in a primary care or similar outpatient setting. A total of 40 rural participants across 20 states, and an additional 59 non-rural participants, participated in year one.

### Purpose of study

The objective of this study was to assess a pilot of the Weitzman APC ECHO Adult Psychiatry Module, which was held from February 2022 to May 2022. A list of topics covered in this module are displayed in Table 1. This study sought to answer the following research question: *In what ways did the Weitzman APC ECHO Adult Psychiatry Module influence learner outcomes and individual practice changes?* This paper aims to fill a gap in the literature by demonstrating the ways in which CE efforts can support rural primary care providers to address the psychiatric needs for their patient population.

**Table 1.** Didactic Topics for APC Adult Psychiatry Module.

Session	Didactic Topic
1	Depression Screening and Initial Evaluation
2	Depression Treatment
3	Anxiety Disorders
4	Post-Traumatic Stress Disorder and Grief Adjustment Disorders
5	Suicidality and Crises Management
6	Psychiatric Medication Management
7	Personality Disorders and Attention-Deficit/Hyperactivity Disorder
8	Social Determinants of Health and Adverse Childhood Experiences

## Materials and methods

### Study design

A one-group repeated measures study design was used to evaluate the influence of the Weitzman APC ECHO Adult Psychiatry Module on learner outcomes. Repeated measures study designs require participants to be exposed to the same intervention (i.e., the APC ECHO module) and assess changes at more than one time point (e.g., pre-post) [13,14]. This study design was selected due to its ability to assess an intervention with a smaller sample to understand participants’ outcomes over time [15]. The study protocol was reviewed by the Community Health Center, Inc. Institutional Review Board and was deemed exempt [IRB Number: 1198; Approval Date: 03/31/2022].

### Study participants and data collection

Participants who registered for the APC ECHO were invited to complete a pre-module survey (*n* = 109), and those that were considered active at the end of the module (i.e., attended at least one session) were invited to complete the post-module survey (*n* = 71).

#### Pre-module survey

Data collection for the pre-module survey occurred over the course of four weeks, with weekly reminder emails to encourage survey completion. A total of 71 responses were received for the pre-module survey (*n* = 71) for a response rate of 64%.

#### Post-module survey

The post-module survey data collection occurred over the course of two weeks, with weekly reminder emails throughout the data collection period. The shortened timeframe for data collection was due to the start date of the next module, which was set to begin two weeks after the end of the first, and to minimize survey fatigue and burden. Thirty participants completed the post-module survey (*n* = 30), for a response rate of 41%.

### Survey tool

Both surveys were administered via Qualtrics Survey Software and were designed based on two frameworks, Moore’s Model of Outcomes Assessment Framework and the Consolidated Framework for Implementation Research [16,17]. The surveys assessed participants’ self-reported knowledge, skills, attitudes, and self-efficacy as they applied to the module’s learning objectives. The pre-module survey collected contextual information about the participants, including their years of experience treating patients with psychiatric conditions, their organizational environment, and the types of patients they serve. Along with changes in the previously mentioned domains, the post-module survey also included questions related to self-reported practice changes resulting from participation in the module.

### Analysis strategy

Participants that completed both the pre-module and post-module surveys were included in the study sample (*n* = 18), encompassing a convenience sample for analysis. Quantitative data analyses were performed using the Stata v17 (College Station, TX). Participant characteristics and individual practice changes were summarized using descriptive statistics (i.e., frequencies and percentages). Open-ended responses to individual practice questions were used to support quantitative data.

Learner outcomes were analyzed to assess differences before and after participation in the ECHO module using repeated measures analysis of variance (ANOVA) with time (pre-module and post-module) as the independent variable. When tests of within-subjects comparisons were significant, indicating a significant change over time, post-hoc predictive margins were performed to compare the pre- and post-module scores and were described using means and standard deviations. Statistical significance was set at p-value <.05.

## Results

### Participant characteristics

Participants were primarily medical providers (*n* = 13; 72.2%), followed by administrative staff (*n* = 3; 16.7%) and clinic leadership (*n* = 2, 11.1%). Among providers that directly work with patients, the majority (*n* = 11, 73.3%) were relatively inexperienced (i.e., less than five years of experience) with providing psychiatric care in a primary care setting; the remainder (*n* = 4, 26.7%) had between 6 and 30 years of experience in psychiatric care.

### Changes in learner outcomes

#### Knowledge changes

A repeated measures ANOVA was performed across the two timepoints (pre-module and post-module) for participants’ perceived changes in learner outcomes; the results are displayed in Table 2. Analysis of knowledge changes across all module objectives revealed statistically significant changes over time (*p* < .01). The post hoc analysis indicated that scores significantly increased from pre-module (21.11 ± 6.99) to post-module (25.08 ± 5.66). For reference, mean scores were reported on a scale of 7 to 35.

**Table 2.** Changes in Learner Outcomes after Participation in APC Adult Psychiatry Module.

Domain	Pre-Module (M+SD)	Post-Module (M+SD)	p-value
Knowledge	21.11 ± 6.99	25.08 ± 5.66	<.01
Self-Efficacy	6.89 ± 3.05	9.78 ± 3.25	<.01
Skills	7.67 ± 4.03	10.06 ± 3.32	<.05
Attitudes	9.22 ± 1.06	9.17 ± 0.99	.805

#### Self-efficacy changes

Participants were asked to indicate their self-efficacy levels related to selecting appropriate first line treatments based on screening and symptomology, utilizing suicidality risk assessment tools, and managing psychiatric crises. Analysis of overall self-efficacy scores indicated a significant change over time (*p* < .01). The post hoc analysis suggested that self-efficacy scores were significantly higher post-intervention (9.78 ± 3.25) compared to the baseline (6.89 ± 3.05). For reference, mean scores were reported on a scale of 3 to 15.

#### Skills changes

Participant skill levels were assessed for the following module objectives: identifying appropriate screening and symptomology instruments for common psychiatric conditions, screening patients for common psychiatric conditions, and managing psychiatric crises. Statistically significant changes were reported across time points (*p* < .05). The post hoc analysis revealed that skills scores significantly increased from baseline (7.67 ± 4.03) to post-intervention (10.06 ± 3.32). For reference, mean scores were reported on a scale of 3 to 15.

#### Attitude changes

Participants identified their attitudes related to care provision for patients with psychiatric conditions. No statistically significant changes were observed across baseline attitude scores (9.22 ± 1.06) to post-intervention scores (9.17 ± 0.99). For reference, mean scores were reported on a scale of 2 to 10.

### Practice Changes

#### Individual practice changes

Participants were also asked how they applied the content learned within the module to their clinic setting (see Table 3); responses were provided on a scale of 1 (strongly disagree) to 5 (strongly agree). Participants indicated the strongest level agreement that they apply best practices learned through the module to all of their patients experiencing psychiatric conditions in a primary care setting (3.96 ± 0.09). In particular, one participant stated, *‘[I now have] greater attention to distress in patients.’* Participants agreed to a slightly lesser extent that they applied new team-based care strategies to their practice as a result of what they learned in the module (3.83 ± 0.12). One participant noted, *‘[I am] continuing to collaborate with other members of the behavioral health team and working to develop work flows to best utilize the talented people on the team.’* Participants had the lowest level agreement that they often shared what they learned from this module with colleagues (3.77 ± 0.23).

**Table 3.** Participant Application of APC Adult Psychiatry Module Content to Clinic Setting.

Statement	M + SD
I apply best practices I have learned through this module to all of my patients experiencing psychiatric conditions in a primary care setting.	3.96 ± 0.09
I have applied new team-based care strategies to my practice as a result of what I learned in this module.	3.83 ± 0.12
I often shared what I learned from this module with colleagues.	3.77 ± 0.23

## Discussion

The purpose of this pilot study was to conduct an assessment of the Weitzman ECHO Advanced Primary Care Adult Psychiatry Module. The findings presented here suggest that the APC Adult Psychiatry Module significantly improved provider knowledge, self-efficacy, and skills across program learning objectives. Findings from this study are consistent with similar WI efforts assessing the impact of ECHO participation on providers’ knowledge and self-efficacy [18,19]. To date, however, this study is WI’s first formal analysis of an ECHO focused on mental health.

Previous efforts have reported results from ECHOs focused on other topic areas. For example, after one year of participation in Weitzman ECHO Pain, primary care providers experienced significant increases in knowledge and self-efficacy over a control group, as well as improved treatment patterns including a decreased use of opioids and increased number of referrals to behavioral health [18]. Similarly, Weitzman ECHO Community Health Workers (CHWs), which provided a learning community specifically for CHWs, increased participant self-efficacy in addressing social determinants of health, with a dose-response relationship evident in CHWs that participated in a higher number of sessions [19]. This alignment in findings suggests that, in some settings, Project ECHO may be an effective CE method for supporting primary care providers’ treatment of psychiatric disorders.

Zhou and colleagues’ 2016 systematic review supported the effectiveness of Project ECHO as an education method for improving provider knowledge and behavior, although none of the 39 studies included in the review reported outcomes on mental health or psychiatry-focused ECHO programs for primary care providers [20]. Since this literature review was published, a small number of more recent studies have emerged reporting provider outcomes associated with mental health Project ECHO programs. For example, ECHO Ontario Mental Health, which consisted of 32, two-hour weekly sessions, reported significant increases in provider knowledge and improvements in self-efficacy that approached significance in a similar pre-post study design [21]. Additionally, the University of New Mexico’s ECHO Institute reported promising results on its Integrated Addictions and Psychiatry ECHO program that met for 2-hour weekly sessions; an analysis of post-session surveys revealed that a majority of providers reported new and broadened knowledge from sessions [22]. Our study strengthens the current literature related to psychiatry-focused ECHO programs by suggesting, even with less frequent, shortened sessions (i.e., twice monthly, 1-hour sessions), learner outcomes will likely improve after participation.

This project is unique and adds to the literature on ECHO programs in several ways. Project ECHO is a relatively fluid model beyond its primary methodologies of brief didactics and interactive case-based learning; other components, including the topics it addresses, audiences it targets, and number and length of sessions are variable across interventions and settings [20,23]. Across its ECHO programs, WI has observed that the time commitment of attending ECHO is one of the most significant barriers to sustained participation over time, and attrition of 50% or more is common. Other ECHO hubs have reported similar findings, including that the time commitment of Project ECHO impacts attendance [24] and that the timing and length of sessions poses a primary barrier to participation [25].

Weitzman ECHO Advanced Primary Care was designed to remain flexible to primary care provider needs. The planning team was cognizant that rural providers’ busy schedules are compounded by healthcare professional shortages in rural areas. To that end, topics were arranged in the modular format described in an attempt to attract a wider audience that otherwise may be deterred by a longer time commitment. Participants are encouraged, but not required, to continue participating across modules and the faculty stays consistent to promote familiarity, rapport, and a sense of community among learners. This study’s significant results suggest that these adaptations compared to a more intensive, weekly ECHO series still produced a feasible and effective ECHO program that improved learner outcomes.

It is also important to situate these results within a larger CE landscape, as ECHO is one tool from a variety of education delivery options. The APC ECHO Adult Psychiatry Module’s structure aligned with recommendations from Cervero and colleagues (2015) on effective mechanisms of CME delivery including its interactivity through case-based discussion and multiple points of exposure over an extended period of time [26]. These recommendations include a fundamental limitation, however. As the majority of efforts to evaluate CME programs are so heterogeneous, it is unknown to what extent different CME formats will be feasible and effective when applied to new content areas for new audiences [26]. Additionally, there have been calls to solve for systematic failures in the CME community to address persisting gaps in best practice care and knowledge through evaluation into learner outcomes [27]. These results respond to this conversation and strengthen the understanding of interactive and extended CE programs as a beneficial mode of delivery.

These results share similarities with other CE programs in psychiatry and mental health topics for a primary care audience, although research in this area is limited. A 2012 review into mental health education programs for a generalist health professional audience found that improvements in participant knowledge were common; fewer studies assessed attitudes and skills [28]. Interestingly, the attitudes domain had the least positive results in this review, and this was the only domain in which APC ECHO did not achieve significant improvements in the present study. The majority of the programs discussed in the review shared delivery similarities with ECHO, including problem or scenario-based learning and active feedback with some didactic content. Of note, the authors predicted that e-learning and experiential methods would become the primary delivery mechanism for this type of education in the future, similar to the ECHO model [28]. Other CE programs in mental health have been offered for mixed primary care audiences including nurses and community health center teams; have covered specialized mental health topics such as co-occurring substance use disorder and pediatric psychopharmacology; and have used a variety of delivery methods such as an intensive one-day course, in-person learning modules delivery at a clinic level, and hybrid approaches combining an initial in-person workshop with virtual, case-based sessions [29–31]. As all of these examples reported positive results across various domains, ECHO is just one approach that may be beneficial.

ECHO offers additional benefits within the context of rural primary care through its accessibility and scalability, which enabled APC ECHO to engage rural providers and those in under-resourced communities dispersed across the U.S. As past studies have found e-learning equivalent in outcomes with in-person CE [32] and the present analysis identified positive learning outcomes, ECHO proved to be a suitable modality for this purpose and audience. It is vital that stakeholders seeking to improve primary care provider practice similarly support and build programs with components that directly address the wants and needs of the audience. Those seeking to improve psychiatric care specifically can be bolstered by the knowledge that many primary care providers have a willingness to learn in this area [9] which may, in part, have also contributed to the practice changes reported by providers after participation in this module.

### Study strengths

This study has several strengths which provide support for future psychiatry-focused virtual CE efforts. Primarily, the study findings observed across self-efficacy and skills demonstrate a more sustainable practice change beyond improved knowledge. Additionally, the study sample was unique in that it included providers that care for patients in a variety of healthcare settings and locations across the U.S., which make the results more generalizable and applicable to various settings. Lastly, the study’s mixed methods and purposeful evaluation presents a holistic understanding of how participants benefited from the learning module beyond learner outcomes to individual practice changes.

### Limitations

This study was limited by its small sample (*n* = 18) that completed both the pre-module and post-module surveys; however, evaluators used a repeated-measures ANOVA design to ensure the analysis performed was robust enough to produce representative results [15]. Additionally, while the study’s focus was on rural primary care providers, the program and sample included providers from sites in non-rural settings and other role types, including behavioral health providers and other care team members. Despite this focus, the module content proved to be impactful for all care team members, indicating its utility across provider types. Lastly, the study’s survey instrument was limited in that it is not a validated survey tool and it assessed participants’ perceived learner outcomes rather than objective measures for each domain. Nevertheless, the survey tool is grounded in the literature on evaluation of CE programs, and similar tools have been used to evaluate other ECHO programs.

### Implications for future efforts

While this pilot study’s preliminary findings are promising, future evaluation efforts could provide stronger support for the impact of the Weitzman ECHO’s APC Adult Psychiatry module as well as other CE trainings for rural primary care providers. In particular, future studies should assess measureable practice changes rather than self-reported changes. Additionally, focusing on either a sample solely of rural medical providers or a comparison across rural versus non-rural providers could help demonstrate the benefit of the program specifically for rural clinicians. Finally, understanding how the module influenced patient outcomes through an assessment of diagnoses and/or management would support a greater understanding of the benefit of the program beyond providers to the patients they serve.

## Conclusions

This pilot study found statistically significant positive changes in knowledge, self-efficacy, and skills as well as individual-level practice changes as a result of participation in the Weitzman ECHO APC Adult Psychiatry Module. The improvement in participant learning outcomes likely supported primary care providers in their provision of behavioral health services for patients who may not have access to specialists in their area. Moreover, results from this study support the growing body of literature on the use of the Project ECHO model as a viable means to increase access to CE and training for providers and address the limited specialists and shortages of behavioral health providers in rural areas of the U.S. With the growing internet access in rural areas, Project ECHO is a scalable and usable tool that rural providers can utilize from any location to improve and standardize psychiatric care in primary care settings. As patient mental health conditions and evidence-based treatment strategies continuously evolve, continued offering of effective CE opportunities focused on mental health will be critical that reflect the growing educational and practice needs of primary care providers.

## References

[1] Rehm J, Shield KD. Global burden of disease and the impact of mental and addictive disorders. Curr Psychiatry Rep. 2019;21(2):1–8. 30729322 10.1007/s11920-019-0997-0

[2] Steel Z, Marnane C, Iranpour C, et al. The global prevalence of common mental disorders: a systematic review and meta-analysis 1980–2013. Int J Epidemiol. 2014;43(2):476–493. DOI:10.1093/ije/dyu038 24648481 PMC3997379

[3] Centers for medicare and medicaid services. Behavioral health. Centers for Medicare and Medicaid Services. Updated November 17, 2020. Accessed September 19, 2020. https://www.cms.gov/outreach-education/american-indianalaska-native/behavioral-health 26110197

[4] U.S. National Institutes of Health. Mental illness. National Institutes Of Health. 2022. Accessed September 19, 2022. https://www-nimh-nih-gov.uc.idm.oclc.org/health/statistics/mental-illness

[5] U.S. Centers for Disease Control and Prevention (CDC). Rates of mental and behavioral health service providers by county. U.S. Centers for Disease Control and Prevention. 2020. Accessed September 19, 2022. https://www.cdc.gov/childrensmentalhealth/stateprofiles-providers.html

[6] Richman EL, Lombardi BM, Zerden LD. Mapping colocation: using national provider identified data to assess primary care and behavioral health colocation. Fam Syst Health. 2020;38(1):16–23. 32202831 10.1037/fsh0000465

[7] Cummings JR, Wen H, Ko M, et al. Geography and the medicaid mental health care infrastructure: implications for health care reform. JAMA Psychiatry. 2013;70(10):1084–1090. 23965816 10.1001/jamapsychiatry.2013.377 PMC4048197

[8] Kyanko KA, Curry LA, Keene DE, et al. Does primary care fill the gap in access to specialty mental health care? A mixed methods study. J Gen Intern Med. 2022;37(7):1641–1647. 34993864 10.1007/s11606-021-07260-z PMC8734538

[9] Fraser K, Oyama O. Knowledge of psychotropics and prescribing preferences of family physicians: a preliminary study. Acad Psychiatry. 2014;37(5):325–328. 10.1176/appi.ap.12090160 24026371

[10] Arora S, Thornton K, Murata G, et al. Outcomes of treatment for hepatitis C virus infection by primary care providers. N Engl J Med. 2011;364(23):2199–2207. DOI:10.1056/NEJMoa1009370 21631316 PMC3820419

[11] University of new Mexico project ECHO. ECHO movement overview. 2022. Accessed Aug 25, 2022. https://hsc.unm.edu/echo/partner-portal/data-marketplace/

[12] Weitzman Institute. Weitzman ECHO. 2022. Accessed August 25, 2022. https://www.weitzmaninstitute.org/education/weitzman-echo/

[13] Field A. Discovering statistics using SPSS. Los Angeles, CA: Sage; 2009.

[14] Leech NL, Barrett KC, Morgan GA. SPSS for intermediate statistics: use and interpretation. 5th ed. New York: Routledge; 2015.

[15] Munro BH. Statistical methods for health care research. 5th ed. Philadelphia, PA, USA: Lippincott, Williams & Wilkins; 2005.

[16] DE M Jr, JSGH G, Gallis HA. Achieving desired results and improved outcomes: integrating planning and assessment throughout learning activities. J Contin Educ Heal Prof. 2009;29(1):1–15. 10.1002/chp.20001 19288562

[17] Damschroder LJ, Aron DC, Keith RE, et al. Fostering implementation of health services research findings into practice: a consolidated framework for advancing implementation science. Implement Sci. 2009;4:50. 19664226 10.1186/1748-5908-4-50 PMC2736161

[18] Anderson D, Zlateva I, Davis B, et al. Improving pain care with project ECHO in community health centers. Pain Med. 2017;18(10):1882–1889. DOI:10.1093/pm/pnx187 29044409 PMC5914304

[19] Damian AJ, Robinson S, Manzoor F, et al. A mixed methods evaluation of the feasibility, acceptability, and impact of a pilot project ECHO for community health workers (CHWs). Pilot Feasibility Stud; 2020;6:132. 10.1186/s40814-020-00678-y PMC7499981 32963804

[20] Zhou C, Crawford A, Serhal E, et al. The impact of project ECHO on participant and patient outcomes: a systematic review. Acad Med. 2016;91(10):1439–1461. 27489018 10.1097/ACM.0000000000001328

[21] Sockalingam S, Arena A, Serhal E, et al. Building provincial mental health capacity in primary care: an evaluation of a project ECHO mental health program. Acad Psychiatry. 2018;42(4):451–457. 28593537 10.1007/s40596-017-0735-z

[22] Komaromy M, Bartlett J, Manis K, et al. Enhanced primary care treatment of behavioral disorders with ECHO case-based learning. Psychiatric Serv. 2017;68(9):873–875. 10.1176/appi.ps.201600471 28806893

[23] Dearing J, Cruz S, Kee K, et al. Project ECHO review and research agenda. Diffusion Associates. 2019. Accessed September 15, 2022. http://www.diffusionassociates.com/pdfs/echo.pdf.

[24] Shimasaki S, Bishop E, Guthrie M, et al. Strengthening the health workforce through the ECHO stages of participation: participants’ perspectives on key facilitators and barriers. J Med Educ Curric. 2019;6:1–8. 10.1177/2382120518820922 PMC6350124 30729170

[25] Shea CM, Gertner AK, Green SL. Barriers and perceived usefulness of an ECHO intervention for office-based buprenorphine treatment for opioid use disorder in North Carolina: a qualitative study. Subst Abuse. 2021;42(1):54–64. 10.1080/08897077.2019.1694617 PMC7274853 31809679

[26] Cervero RM, Gaines JK. The impact of CME on physician performance and patient health outcomes: an updated synthesis of systematic reviews. J Contin Educ Health Prof. 2015;35(2):131–138. 26115113 10.1002/chp.21290

[27] Nissen SE. Reforming the continuing medical education system. JAMA. 2015;313(18):1813–1814. 25965221 10.1001/jama.2015.4138

[28] Brunero S, Jeon YH, Foster K. Mental health education programmes for generalist health professionals: an integrative review. Int J Ment Health Nurs. 2012;21(5):428–444. 22500589 10.1111/j.1447-0349.2011.00802.x

[29] Jenkins E, Currie LM, Hirani S, et al. Enhancing nurses’ capacity to provide concurrent mental health and substance use disorder care: a quasi-experimental intervention study. Nurse Educ Today. 2022;117:105483. 35908405 10.1016/j.nedt.2022.105483 PMC9970037

[30] Khenti A, Thomas FC, Mohamoud S, et al. Mental health and addictions capacity building for community health centres in Ontario. Can Fam Physician. 2017;63(10):e416–424. 29025818 PMC5638489

[31] McCaffrey ESN, Chang S, Farrelly G, et al. Mental health literacy in primary care: Canadian Research and Education for the Advancement of Child Health (CanREACH). Evid Based Med. 2017;22(4):123–131. 28735276 10.1136/ebmed-2017-110714 PMC5537558

[32] Samuel A, Cervero RM, Durning SJ, et al. Effect of continuing professional development on health professionals’ performance and patient outcomes: a Scoping review of knowledge syntheses. Acad Med. 2021;96(6):913–923. 33332905 10.1097/ACM.0000000000003899

